# The Complex Effect of Food Matrix Fat Content on Thermal Inactivation of *Listeria monocytogenes*: Case Study in Emulsion and Gelled Emulsion Model Systems

**DOI:** 10.3389/fmicb.2019.03149

**Published:** 2020-01-22

**Authors:** Davy Verheyen, Marlies Govaert, Ti Kian Seow, Jonela Ruvina, Vivek Mukherjee, Maria Baka, Torstein Skåra, Jan F. M. Van Impe

**Affiliations:** ^1^BioTeC+ – Chemical and Biochemical Process Technology and Control, KU Leuven, Ghent, Belgium; ^2^Center of Excellence Optimization in Engineering, KU Leuven, Ghent, Belgium; ^3^Flemish Cluster Predictive Microbiology in Foods (CPMF^2^), Ghent, Belgium; ^4^Nofima, Stavanger, Norway

**Keywords:** thermal inactivation kinetics, predictive microbiology, *Listeria monocytogenes*, fat content, food microstructure

## Abstract

Previous studies on the influence of food matrix fat content on thermal inactivation kinetics of food pathogens have shown contradictory results due to the combined influence of fat content and other factors such as composition. Therefore, thermal inactivation of *Listeria monocytogenes* at 59, 64, and 69°C was systematically investigated in emulsion and gelled emulsion food model systems with various fat content (1, 5, 10, and 20%), such that the effect of fat content was isolated. Thermal conductivity and rheological properties of the model systems were quantified, as well as the effect of these properties on the thermal load of the model systems. Thermal conductivity was complexly related to fat content, the nature of the food matrix (i.e., viscous or gelled), and temperature. For the emulsions, the consistency index *K* increased with increasing fat content, while the flow behavior index *n* followed the opposite trend. For the gelled emulsions, the storage modulus *G*′ was always larger than the loss modulus *G*″ (i.e., measure of elastic and viscous properties, respectively). The phase angle δ [i.e., arctan (*G*″/*G*′)] was proportional with fat content, but this relation became more complex at higher temperatures. The thermal load of the model systems was not largely affected by food matrix fat content. Thermal inactivation of *L. monocytogenes* was investigated by means of the maximum specific inactivation rate *k*_max_, log reductions, and sublethal injury (SI). Both for emulsions and gelled emulsions, *k*_max_ decreased with increasing fat content below approximately 60°C, while a more complex behavior was observed at higher temperatures. In the emulsions, log reductions were considerably lower (i.e., 2–3 log) at 1% fat than in systems with higher fat content. In the gelled emulsions, log reductions generally decreased with increasing fat content. SI decreased with increasing fat content, both in emulsions and gelled emulsions. In conclusion, the inactivation rate (i.e., *k*_max_) of *L. monocytogenes* was affected by a complex relation between food matrix fat content, thermal conductivity, rheological properties, and inactivation temperature. Due to the small scale of the model systems, differences in *k*_max_ did not directly affect the final log reductions in a similar fashion.

## Introduction

Thermal processing remains one of the most used methods in food industry to ensure the microbial safety and extend the shelf life of many different food products (Pratap Singh et al., 2018). It has been shown that the presence of fat droplets inside food products significantly affects microbial inactivation kinetics during thermal processes (Verheyen et al., 2019a). Acquiring fundamental knowledge about the influence of fat content on thermal inactivation kinetics would be beneficial to acquire more insight into the influencing mechanisms of the presence of fat droplets on thermal inactivation of bacteria.

The effect of fat content on the thermal inactivation of food pathogens has been investigated to some extent in the past, but different studies show contradictory results. Increasing the fat content of food products resulted in an increase (Fain et al., 1991; Ahmed et al., 1995; Chhabra et al., 1999; Juneja et al., 2001) or decrease (Schultze et al., 2007; Oliveira et al., 2018) of the resistance to thermal inactivation of submerged bacteria in the foods, depending on the specific case study. In other studies, no significant effect of fat content on thermal inactivation kinetics was observed (Kotrola and Conner, 1997; Stoltenberg et al., 2006; Byelashov et al., 2010; Kim and Kang, 2015). Apart from the influence of bacterial species and strains, differences in inactivation kinetics could be caused, at least in part, by variations in compositional and physicochemical factors due to the use of real food products in the aforementioned studies. In addition, these microbiological studies did not take into account that food matrix fat content also influences heat transfer dynamics inside the food product during thermal processing, both when considering conductive and convective heat transfer (Cordioli et al., 2016; Phinney et al., 2017).

Recently, researchers started using artificial model systems with various microstructures [e.g., liquids, aqueous gels, oil-in-water emulsions, water-in-oil emulsions, gelled emulsions, and surfaces, according to the classification of Wilson et al. (2002)] to acquire more insight into the effect of food microstructural aspects on microbial growth dynamics (Baka et al., 2016, 2017a,b). Verheyen et al. (2018) further improved this concept by developing a set of model systems exhibiting minimal variations in compositional and physicochemical factors among the different systems. This method enables the isolation of certain microstructural aspects such as the nature of the food matrix (e.g., viscous or gelled) and the presence of fat droplets. In relation to the latter, the effect of food matrix fat content can also be isolated. Hence, emulsion and gelled emulsion model systems, prepared according to this approach, could be used to study the influence of food matrix fat content on thermal inactivation kinetics of bacteria.

In this study, the effect of food matrix fat content on thermal inactivation kinetics of *Listeria monocytogenes* was investigated using small-scale (i.e., 1 mL) artificial food model systems. Model system composition was based on processed fish products containing a certain amount of fat (e.g., fish paté, fish salad, fish sausage) and major food microstructural elements of such food products (e.g., fat droplets, a viscoelastic matrix) were represented in the systems. In order to isolate the effect of fat content on microbial behavior, model systems with minimal variation in compositional and physicochemical factors were used, i.e., emulsions and gelled emulsions with fat contents of 5, 10, and 20% (Verheyen et al., 2018), and the obtained results were compared to those for systems containing 1% fat (Verheyen et al., 2019a). These systems spanned a fat content range of 1–20% which is relevant for a plethora of different processed fish products (Aquerreta et al., 2002; Raju et al., 2003; Echarte et al., 2004; Manthey-Karl et al., 2014). The model systems were characterized for their thermal conductivity and rheological properties to investigate possible effects of fat content on conductive and convective heat transfer inside the model systems. Inactivation experiments were conducted at temperatures of 59, 64, and 69°C, representing a temperature range relevant for mild thermal treatments of processed fish products. Inactivation rates, log reductions, and sublethal injury (SI) induced in the cells were compared among systems with different fat content. Finally, findings were compared to those from studies which investigated the influence of food matrix fat content on *L. monocytogenes* inactivation in real food products.

## Materials and Methods

### Model System Preparation

Emulsion (Em) and gelled emulsion (GE) model systems with different fat content (i.e., 1, 5, 10, and 20%) were prepared as described by Verheyen et al. (2018). The systems were divided over small vials (4 mL, 45 × 14.7 mm, BGB Analytik Benelux B.V., Harderwijk, Netherlands), to a volume of 1 mL of viscous or gelled medium per vial. Table 1 shows the composition of the different model systems. A more detailed overview of the physicochemical properties and other characteristics of the model systems is provided by Verheyen et al. (2018, 2020). From this point on, emulsions and gelled emulsions with different fat content were defined as Em1, Em5, Em10, Em20, GE1, GE5, GE10, and GE20, respectively.

**TABLE 1:** Composition of the emulsion and gelled emulsion model systems with different fat content (Verheyen et al., 2020).

Ingredients (% w/w)	Emulsion				Gelled emulsion			
		
	1%	5%	10%	20%	1%	5%	10%	20%
Fish protein	5.00	5.00	5.00	5.00	5.00	5.00	5.00	5.00
Alginate	3.00	3.00	3.00	3.00	3.00	3.00	3.00	3.00
NaCl	0.95	0.90	0.84	0.74	0.94	0.89	0.84	0.73
CaCO_3_	/	/	/	/	0.40	0.38	0.36	0.31
GDL^1^	/	/	/	/	0.95	0.90	0.84	0.74
Sunflower oil	1.00	5.00	10.00	20.00	1.00	5.00	10.00	20.00
Tween 80	0.10	0.35	0.35	0.35	0.10	0.35	0.35	0.35
Span 80	0.20	0.65	0.65	0.65	0.20	0.65	0.65	0.65
Xanthan gum	0.50	0.50	0.50	0.50	/	/	/	/
Distilled H_2_O	89.25	84.6	79.66	69.76	88.41	83.83	78.96	69.22

*^1^D-(+)-gluconic acid δ-lactone.*

### Thermal Conductivity

In order to investigate the influence of food matrix fat content on conductive heat transfer, thermal conductivity was quantified in the emulsion and gelled emulsion model systems with different fat content (i.e., 1, 5, 10, and 20%). Thermal conductivity was measured at 59 and 69°C (i.e., the minimum and maximum temperature used in the current study) by a line heating source probe and instrument KD2 (Decagon devices Inc., Pullman, WA, United States). Samples consisting of 50 g per model system were used. Experiments were conducted independently in triplicate.

### Rheological Properties

In order to investigate the influence of food matrix fat content on convective heat transfer, rheological properties of the emulsions and gelled emulsions with different fat content (i.e., 1, 5, 10, and 20%) were quantified. The rheological properties of the model systems were characterized using a Discovery Hybrid Rheometer (DHR-2, TA Instruments, New Castle, DE, United States), equipped with a 40-mm parallel plate system, consisting of a crosshatched upper and lower plate. The gap size between the two plates was set at 500 μm for viscous systems and at 1000 μm for gelled systems. Temperature control was regulated by means of a Peltier temperature control system and upper heated plate.

The emulsions were characterized by means of their steady-state behavior at 20.0, 32.5, 45.0, 57.5, and 70.0°C, based on the protocol of Verheyen et al. (2018). Model systems were prepared approximately 24 h prior to the rheological measurements and stored at 10°C. A delay of 2 min was set after sample loading in order to allow temperature calibration and relaxation of stresses induced during the loading procedure. A sample of 1.6 mL of each model system was carefully transferred to the bottom plate. The shear rate was increased linearly from 0.01 to 50.00 Hz, and the corresponding shear stress was evaluated after the sample reached steady-state. The relationship between the shear stress and shear rate was characterized by Equation 1, which represents the power law model (Reiner, 1926).

(1) $$\text{τ=}\,K\cdot {\dot{\gamma }}^{n}$$

With τ (Pa), the shear stress; $\dot{\gamma }$ (1/s), the shear rate; *K* (Pa/s), the consistency index; and *n* (-), the flow behavior index representing the extent of deviation from Newtonian behavior. Both model parameters (i.e., *K* and *n*) were estimated by fitting a power function to the experimental data using Microsoft Excel (Microsoft Corporation, WA, United States). This procedure was conducted for each of the five temperatures, resulting in a characterization of the rheological behavior of the emulsions over the relevant temperature range. Experiments were conducted independently in triplicate.

Gelled systems (i.e., gelled emulsions containing 1, 5, 10, and 20% fat) were characterized by means of a temperature ramp procedure over a temperature range from 20 to 70°C. Approximately 24 h prior to the rheological measurements, gelled systems were prepared in round Petri dishes (55 × 12 mm, Anicrin S.R.L., Scorzè, Italy) by allowing 6 mL of viscous medium to solidify in the plates (Verheyen et al., 2018), and stored at 10°C. A delay of 2 min was set after sample loading onto the rheometer. The ramp rate, oscillatory stress and angular frequency were set to 2°C/min, 10 Pa, and 1 Hz, respectively. Experiments were conducted independently in triplicate.

### Thermal Inactivation Experiments

A strain cocktail of three *L. monocytogenes* strains, acquired from the BCCM/LMG bacteria collection of Ghent University in Belgium, was used, i.e., LMG 23773, LMG 23774 (both isolated from smoked salmon), and LMG 26484 (isolated from tuna salad). The preparation of precultures and the inoculation of the model systems was performed as described in Verheyen et al. (2019a). Briefly, a purity plate was prepared for each strain by spreading a loopful of stock culture onto a BHI Agar plate [1.4% (w/v), Agar Technical No3, Oxoid Ltd., Basingstoke, United Kingdom], which was incubated at 30°C for 24 h. Two consecutive 24 h-precultures (incubated at 30°C) in BHI were prepared for each strain individually, starting from one colony of the respective purity plate. Equal volume aliquots of (second) precultures of the three strains were mixed, resulting in a stationary phase *L. monocytogenes* strain cocktail with a cell density of approximately 10^9^ CFU/mL. Model systems were homogeneously inoculated to a cell density of 10^8^–10^9^ CFU/mL prior to the division over the small vials. Inoculated model systems were stored at 10°C for approximately 16 h prior to the respective inactivation experiments in order to allow the gelled systems to solidify.

Thermal inactivation experiments and sample processing were conducted as described by Verheyen et al. (2019a). In short, vials containing the model systems were immersed in water baths at a constant temperature of 59, 64, or 69°C, removed at different time intervals, and stored in ice-water prior to further processing. Serial dilutions (i.e., prepared using a 0.90% w/v NaCl solution) were plated on BHI agar and PALCAM agar (VWR Chemicals, Leuven, Belgium), employing the drop technique (Herigstad et al., 2001), with enumeration after approximately 30 h at 30°C. Cell counts were considered significant when at least 10 colonies per two drops of 100 μL were counted on the agar plates for the undiluted sample, resulting in a detection limit (DL) of 1.7 log (CFU/mL) for emulsions and 2.3 log (CFU/mL) for gelled emulsions. The higher DL for gelled emulsions was caused by an extra dilution step needed to dissolve the gels during sample processing. Replicates for which colony counts below the DL were detected, a situation which only occurred in the emulsions systems, were taken into account for the model fit as such. Replicates for which no colonies were detected in the undiluted sample were assumed to be equal to the average of the DL and zero, i.e., 0.85 log (CFU/mL) for emulsions. In both cases, however, results were critically evaluated. All experiments were performed independently in triplicate.

### Temperature Measurements

For each model system at each temperature, the core temperature during the thermal inactivation experiments was recorded every 2 s using a Testo 176 T4 temperature datalogger connected to two TC Type K temperature probes and data was acquired using ComSoft Basic 5 logger software (Testo SE & Co. KGaA, Lenzkirch, Germany). The thermal load to which the model systems were exposed during the different thermal treatments was calculated by integrating the obtained temperature curves over time. Temperature profiles were recorded independently in triplicate.

### Estimation of Inactivation Kinetics

The inactivation model of Geeraerd et al. (2000), extended with a Bigelow-type temperature dependency (Garre et al., 2017), was fitted to the experimental data on BHI agar (i.e., providing inactivation dynamics for the total population of uninjured and injured cells). The inactivation rate was set to zero for temperatures lower than 50°C, since these temperatures do not result in *L. monocytogenes* inactivation (Valdramidis et al., 2006). Model parameters were estimated from the set of experimental data via the minimization of the sum of squared errors, using the lsqnonlin routine of the Optimization Toolbox of MatLab^®^ version R2018b (The Mathworks Inc., Natick, United States). For each model system, the differential equations of the inactivation model were solved simultaneously (i.e., one-step procedure) for the inactivation data at 59, 64, and 69°C, using the MatLab solver ode45. Standard errors of parameter estimates were calculated from the Jacobian matrix. A one-step procedure was used in order to cope with the dynamic temperature profiles *T*(*t*) which are inseparably linked to the heat transfer characteristics in non-capillary model systems. In this regard, inactivation experiments in the current study involved come-up times (i.e., time necessary for the model systems to reach the final temperature) of more than 2 min. Furthermore, the one-step fitting procedure avoids extra errors which are introduced in two-step procedures by the use of estimated kinetic parameters for the fitting procedure (Van Derlinden et al., 2008).

The model of Geeraerd et al. (2000) and the (conditional) Bigelow-type relation (Garre et al., 2017) are represented by Eqs. 2 and 3, respectively.

(2) $$\frac{\text{d}\,N\,\left(t\right)}{\text{d}\,t}\,\text{=}-k_{\text{max}}\,\left(1-\frac{N_{\text{res}}}{N\,\left(t\right)}\right)\,N\,\left(t\right)$$

(3) kmax⁢(T⁢(t))⁢=⁢{kmax⁢(Tref)⋅10T⁢(t)-Trefz,i⁢f⁢T≥50⁢C∘0,if⁢T<50⁢C∘

With *N* (CFU/mL), the cell density at time *t*; *N*_*res*_ (CFU/mL), the residual cell density; *k*_max_ (1/min), the maximum specific inactivation rate; *T*(*t*) (°C), the core-temperature of the model system; *T*_*ref*_ (°C), the reference temperature; *k*_max_(*T*_ref_) (1/min), the maximum specific inactivation rate at the reference temperature; and *z* (°C), representing the sensitivity of *k*_max_ to temperature changes. A reference temperature *T*_ref_ equal to 64°C was selected and core-temperatures of the model systems were calculated as the average of the independent temperature measurements. Log reductions were calculated based on the modeled values for the initial cell density *N*_0_ and the residual cell population *N*_*res*_.

### Sublethal Injury Assessment

The evolution of SI was calculated as described by Verheyen et al. (2019a). Briefly, SI was calculated for each replicate at each time point, using Eq. 4 (Busch and Donnelly, 1992), and a third-degree polynomial was fitted to all obtained datapoints for each condition. BHI and PALCAM agar (VWR Chemicals, Leuven, Belgium) were used as a non-selective (i.e., representing the total population of injured and uninjured cells) and selective (i.e., representing the population of injured cells) medium, respectively.

Sublethal injury was assumed to be equal to zero when counts on PALCAM were higher than those on BHI, and datapoints for which the counts on BHI were below the DL were omitted.

(4) $$\text{SI=}\frac{\log\left(\text{CFU}\right)\,\mathrm{on}\,\mathrm{BHI}\,\mathrm{agar}-\text{log}\,\left(\text{CFU}\right)\,\mathrm{on}\,\mathrm{PALCAM}\,\mathrm{agar}}{\log\left(\text{CFU}\right)\,\mathrm{on}\,\mathrm{BHI}\,\mathrm{agar}}\cdot 100\%$$

In order to quantify the total amount of SI over the duration of the thermal treatment, the Time-averaged Injured Cells Coefficient (TICC) was calculated according to the formula of Miller et al. (2006), as described in Eq. 5.

(5) $$\text{TICC}=\frac{\int_{0}^{t_{f}}\text{SI}\,\left(t\right)\,\text{d}\,t}{t_{f}}$$

With *t* (min), the treatment time; *t*_*f*_ (min), the total duration of the thermal treatment; and SI (−), the percentage of sublethal injury according to the fitted polynomial.

### Statistical Analysis

Significant differences between model parameters were determined using analysis of variance (ANOVA, single variance) test at a 95.0% confidence level (α = 0.05). Fisher’s Least Significant Difference test was used to distinguish which means were significantly different from others. Standardized skewness and standardized kurtosis were used to assess if data sets came from normal distributions. The analyses were performed using Statgraphics Centurion XVII Package (Statistical Graphics, Washington, United States). Test statistics were regarded as significant when *P* ≤ 0.05.

## Results and Discussion

### Thermal Conductivity

Table 2 provides an overview of the thermal conductivity values for the emulsions and gelled emulsions with different fat content, measured at 59 and 69°C. In the emulsions at 59°C, an increase in fat content from 1 to 5% led to a significant decrease in thermal conductivity. Between 5 and 20% fat content, thermal conductivity increased with increasing fat content. In the emulsions at 69°C, an increase in fat content from 1 to 5% also led to a significant decrease in thermal conductivity, but no significant differences were observed among Em5, Em10, and Em20. In the gelled emulsions at 59°C, an increase in fat content from 1 to 5% led to a significant increase in thermal conductivity, while no significant differences were observed among GE5, GE10, and GE20. In the gelled emulsions at 69°C, thermal conductivity decreased with increasing fat content, although no significant differences were observed between GE1 and GE5.

**TABLE 2:** Thermal conductivity (W/mK) of the emulsion and gelled emulsions model systems with different fat content (i.e., 1, 5, 10, and 20%), obtained at 59 and 69°C.

	Emulsions		Gelled emulsions	
		
Fat content (%)	59°C	69°C	59°C	69°C
1	0.45 ± 0.03^C^	0.60 ± 0.02^B^	0.53 ± 0.02^A^	0.56 ± 0.03^C^
5	0.35 ± 0.03^A^	0.46 ± 0.04^A^	0.60 ± 0.01^B^	0.56 ± 0.02^C^
10	0.40 ± 0.00^B^	0.48 ± 0.04^A^	0.60 ± 0.01^B^	0.51 ± 0.01^B^
20	0.42 ± 0.00^BC^	0.48 ± 0.04^A^	0.60 ± 0.01^B^	0.44 ± 0.02^A^

*Three independent replicates were conducted. For the different fat contents, values bearing different uppercase letters are significantly different (*P* ≤ 0.05).*

In general, results of the current study are more complex than the trend of linearly decreasing thermal conductivity with increasing food matrix fat content which has been commonly reported for both viscous (Pereira et al., 2013) and gelled emulsion (Baghe-Khandan et al., 1982; Tavman and Tavman, 1999) food products. Solely based on model system composition and following the modeling approach of Phinney et al. (2017), thermal conductivity would decrease from approximately 0.63 W/(mK) at 1% fat to approximately 0.52 W/(mK) at 20% fat, almost independently of temperature (results not shown). Consequently, thermal conductivity values cannot be directly linked to the differences in thermal conductivity between sunflower oil and water (i.e., 0.168–0.162 and 0.598–0.660 W/(mK), respectively) over the relevant temperature range (Turgut et al., 2009; Sharqawy, 2013), implying that the thermal conductivity of the model systems was affected by phenomena occurring during the (gelled) emulsion preparation process, e.g., the homogenization step at 22 000 rpm.

In order to elucidate the effect of the presence of an “emulsion structure,” thermal conductivity values were compared to those obtained for similar systems without fat, as reported by Erdogdu et al. (2018). For the emulsions, results were compared to those in a liquid system which only differed from Em1 by the absence of 1% sunflower oil and 0.30% emulsifiers (i.e., Tween 80 and Span 80). Both at 59 and 69°C, the thermal conductivity in this liquid system (i.e., 0.60 ± 0.04 and 0.67 ± 0.03 W/mK, respectively) was significantly higher than in Em1. Hence, it can be concluded that imposing an “emulsion structure” results in significantly lower thermal conductivity values, although the extent of this decrease is less evident at higher temperatures. In addition, increasing the fat content from 1 to 5% exerts a larger effect on thermal conductivity than relatively larger fat content differences between 5 and 20% fat. In this regard, protein aggregate rearrangement during the homogenization process might have resulted in a steady charge distribution which was not largely affected by differences in fat content in the range from 5 to 20% fat (Dybowska, 2011). For the gelled emulsions, results were compared to those in an aqueous gel which also only differed from GE1 by the absence of sunflower oil and emulsifiers. At 59°C, the thermal conductivity in the aqueous gel (i.e., 0.60 ± 0.04 W/mK) was significantly higher than in GE1. At 69°C, on the other hand, no significant differences in thermal conductivity were observed between the aqueous gel (i.e., 0.55 ± 0.02 W/mK) and GE1. Consequently, thermal conductivity of the gelled systems seemed to increase when imposing an “emulsion structure,” although only at 59°C. This effect at 59°C is opposite to the effect observed for the viscous emulsions. Similar to the viscous emulsions, however, increasing the fat content from 1 to 5% exerts a larger effect on thermal conductivity than relatively larger fat content differences between 5 and 20%. At 69°C, on the other hand, differences in thermal conductivity seem more closely related to the model system composition, i.e., thermal conductivity was decreasing with increasing fat content.

It can therefore be concluded that the effect of food matrix fat content on thermal conductivity is intertwined with the nature of the food matrix (i.e., viscous or gelled) as well as temperature. Microstructural differences caused by differences in fat content during the (gelled) emulsion preparation process seem to exert a larger influence on thermal conductivity than the compositional differences. In addition, these microstructural aspects are significantly affected by temperature.

### Rheological Properties

For the emulsions with different fat content (i.e., 1, 5, 10, and 20%), the parameters *K* and *n* of the power law model (Reiner, 1926), over a temperature range of 20 to 70°C are presented in Table 3. On the one hand, the consistency index *K* can be interpreted as a measure for the viscosity of non-Newtonian fluids. The flow behavior index *n*, on the other hand, quantifies the extent of deviation from Newtonian behavior (i.e., *n* = 1 for Newtonian fluids), with a lower value representing a larger deviation. The consistency index *K* followed a similar trend at each temperature, i.e., *K* (Em1) ≤ *K* (Em5) ≤ *K* (Em10) ≤ *K* (Em20). Differences were not statistically significant at all temperatures, but *K* was always the lowest in Em1 and the highest in Em20. These findings correspond to the trend of increasing viscosity with increasing fat content, which is commonly reported in literature (Wendin and Hall, 2001). Results for the flow behavior index *n* indicate that all emulsions exhibit pseudo-plastic behavior (i.e., *n* < 1), as already reported for Em1 at 20°C (Verheyen et al., 2018). Concerning the influence of fat content on the rheological properties of the emulsions, the flow behavior index *n* also followed a similar trend at each temperature, i.e., *n* (Em1) ≥ *n* (Em5) ≥ *n* (Em10) ≥ *n* (Em20). Hence, the rheological behavior of the emulsions deviated more from Newtonian behavior (i.e., increased pseudo-plastic behavior) with increasing fat content. An increase in *K* and decrease in *n* with increasing fat content were also observed by Vélez-Ruiz et al. (2005). This phenomenon may be linked to interactions between the xanthan gum network and the emulsion droplets, stabilizing the emulsion by means of hydrogen bonding (Lim et al., 1984; Ma and Barbosa-Cánovas, 1995).

**TABLE 3:** Consistency index *K* and flow behavior index *n* of the emulsions with different fat content (i.e., 1, 5, 10, and 20%), according to the power law model (Reiner, 1926).

T (°C)	*K* (Pa/s)				*n* (-)			
		
	1%	5%	10%	20%	1%	5%	10%	20%
20.0°C	1.054 ± 0.140^A^	1.483 ± 0.159^A^	1.786 ± 0.017^B^	5.477 ± 0.528^C^	0.766 ± 0.017^C^	0.787 ± 0.004^C^	0.646 ± 0.002^B^	0.528 ± 0.019^A^
32.5°C	0.623 ± 0.060^A^	0.927 ± 0.088^AB^	1.529 ± 0.090^B^	4.243 ± 0.573^C^	0.774 ± 0.010^C^	0.779 ± 0.005^C^	0.653 ± 0.003^B^	0.512 ± 0.007^A^
45.0°C	0.372 ± 0.043^A^	0.671 ± 0.164^A^	1.226 ± 0.226^A^	3.631 ± 0.671^B^	0.808 ± 0.004^C^	0.807 ± 0.010^C^	0.655 ± 0.001^B^	0.491 ± 0.015^A^
57.5°C	0.309 ± 0.001^A^	0.481 ± 0.116^A^	1.111 ± 0.126^B^	2.993 ± 0.262^C^	0.823 ± 0.014^C^	0.782 ± 0.010^C^	0.649 ± 0.032^B^	0.489 ± 0.017^A^
70.0°C	0.205 ± 0.005^A^	0.303 ± 0.003^A^	0.973 ± 0.026^B^	1.937 ± 0.082^C^	0.791 ± 0.010^D^	0.730 ± 0.026^C^	0.619 ± 0.009^B^	0.554 ± 0.002^A^

*Three independent replicates were conducted. For the different model systems at one temperature, values bearing different uppercase letters are significantly different (*P* ≤ 0.05).*

**FIGURE 1:**
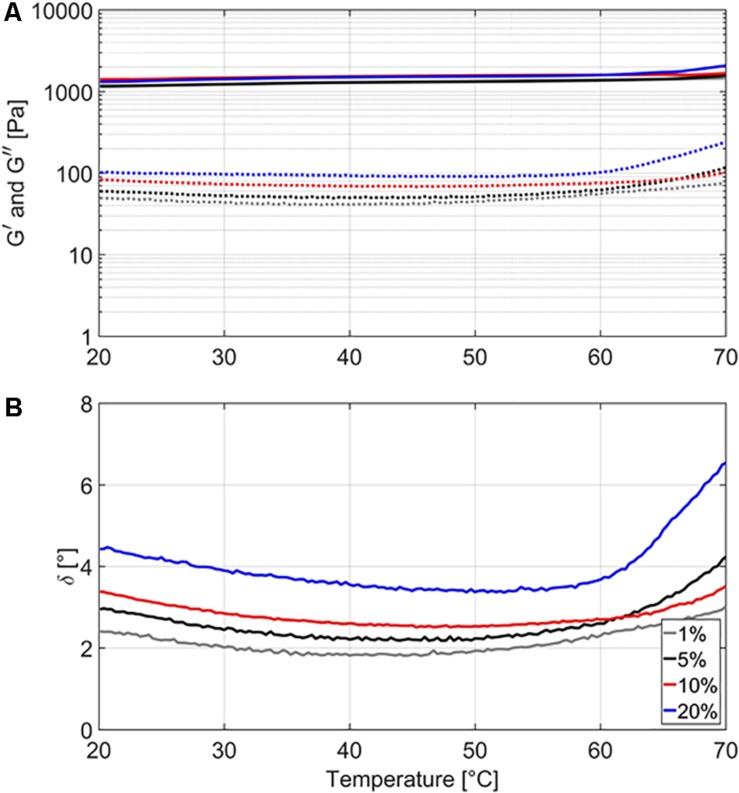
Storage modulus *G****′*** (**A**, full lines), loss modulus *G****″*** (**A**, dashed lines), and phase angle δ **(B)** of the gelled emulsions with different fat content (i.e., 1, 5, 10, and 20%), measured over a temperature range from 20 to 70°C. Three independent replicates were conducted.

For the gelled emulsions with different fat content (i.e., 1, 5, 10, and 20%), Figure 1 illustrates the evolution of the storage modulus *G*′, the loss modulus *G*″, and the phase angle δ over a temperature range of 20–70°C. The storage modulus *G*′ can be interpreted as a measure of the elastic properties of a food product, while the loss modulus *G*″ can be interpreted as a measure of the viscous properties. The phase angle δ is calculated as arctan (G*″*/G*′*) and signifies the ratio of the viscous to the elastic portion of the deformation behavior, with δ = 0°corresponding to elastic behavior, δ = 90° to viscous behavior, and 0 < δ < 90 to viscoelastic behavior (Tabilo-Munigaza and Barbosa-Cánovas, 2005). Storage moduli *G*′ increased from 1171 to 1458 Pa for GE1, from 1160 to 1579 Pa for GE5, from 1408 to 1672 Pa for GE10, and from 1312 to 2073 Pa for GE20. Loss moduli *G*″ first decreased, then went through a minimum at a temperature between 30 and 50°C, and then increased again. More specifically, loss moduli *G*″ had starting, minimum, and maximum values of 49, 40, and 77 Pa for GE1, 60, 49, and 116 Pa for GE5, 83, 68, and 103 Pa for GE10, and 101, 89, and 237 Pa for GE20. Since *G*′ was always much greater than *G*″, all gelled emulsions exhibited solid-like behavior (i.e., strong gels), indicating that deformations are recovered elastically rather than by viscous dissipation (Lopes da Silva and Rao, 2007). The phase angle δ gradually decreased with increasing temperature in all model systems until a temperature of approximately 50°C was reached. Afterward, δ started to increase with increasing temperature, with this evolution being the most prominent for GE5 and GE20. Up until a temperature of approximately 63°C, δ was higher for systems with higher fat content, implying a more viscous behavior at higher fat content. At higher temperatures, however, the fast increase in δ of GE5 made this system behave more viscous than GE10. The relation between food matrix fat content and gel strength of gelled emulsions is known to depend on the specific microstructural aspects of the food product of interest. For example, gel strength increases with increasing fat content in mayonnaise (Ma and Barbosa-Cánovas, 1995), while the opposite is observed in Cheddar cheese and certain dairy desserts (Guinee et al., 2000; Arancibia et al., 2015).

### Thermal Load

In order to investigate the influence of food matrix fat content on heat transfer, the thermal load to which the model systems were exposed during the thermal treatments was quantified, as presented in Table 4. Values for systems containing 1% fat, as calculated from the data of Verheyen et al. (2019a), were also included. In the emulsions, no significant differences in thermal load were observed among the different systems at 64 and 69°C. At 59°C, the thermal load (i) increased when increasing the fat content from 1 to 5%, (ii) remained constant when increasing the fat content to 10%, and (iii) decreased again when increasing the fat content to 20%. In the gelled emulsions, no significant differences in thermal load were observed among the different systems at 59°C. At 64 and 69°C, thermal load increased when increasing the fat content from 1 to 5% and started to decrease again at a higher fat content dependent on the treatment temperature, i.e., 20 and 10% fat at 64 and 69°C, respectively.

**TABLE 4:** Thermal load to which the emulsion and gelled emulsion model systems with different fat content (i.e., 1, 5, 10, and 20%) were exposed over the course of the inactivation treatments at the different temperatures (i.e., 59, 64, and 69°C).

	Emulsions			Gelled emulsions		
		
Fat content (%)	59°C	64°C	69°C	59°C	64°C	69°C
1	104440 ± 174^B1^	56255 ± 135^A1^	39830 ± 175^A1^	104953 ± 190^A1^	56015 ± 59^A1^	39770 ± 91^A1^
5	104955 ± 180^BC^	56380 ± 168^A^	39752 ± 90^A^	104963 ± 85^A^	56215 ± 46^C^	39915 ± 45^B^
10	105171 ± 119^C^	56556 ± 134^A^	39984 ± 70^A^	105075 ± 204^A^	56204 ± 57^C^	39789 ± 24^A^
20	104829 ± 145^B^	56516 ± 305^A^	39693 ± 63^A^	105009 ± 150^A^	56128 ± 20^B^	39731 ± 72^A^

*^1^Calculated based on data from Verheyen et al. (2019a). Three independent replicates were conducted. For the different model systems at one temperature, values bearing different uppercase letters are significantly different (*P* ≤ 0.05).
*

For the emulsions, a comparison of the thermal load values to thermal conductivity values and rheological properties of the model systems demonstrates that differences in thermal load (if present) were not due to differences in conductive or convective heat transfer. Similar conclusions can be drawn for the gelled emulsions, even though convective heat transfer is in any case impossible in those systems due to their solid-like behavior. However, observed significant differences among the systems with different fat content at a certain temperature were rather small, i.e., relative differences of maximum 0.7%. Therefore, the fat content of the model systems probably exerted no significant influence on heat transfer dynamics at this small scale (i.e., 1 mL of model system). Similar conclusions were drawn by Kim and Kang (2015) in emulsion samples of 25 mL containing 3, 7, and 10% fat. In future studies, the used model systems can be scaled up to investigate the influence of fat content on heat transfer and link this influence with the direct effect on the microorganisms.

### Thermal Inactivation of *Listeria monocytogenes*

**FIGURE 2:**
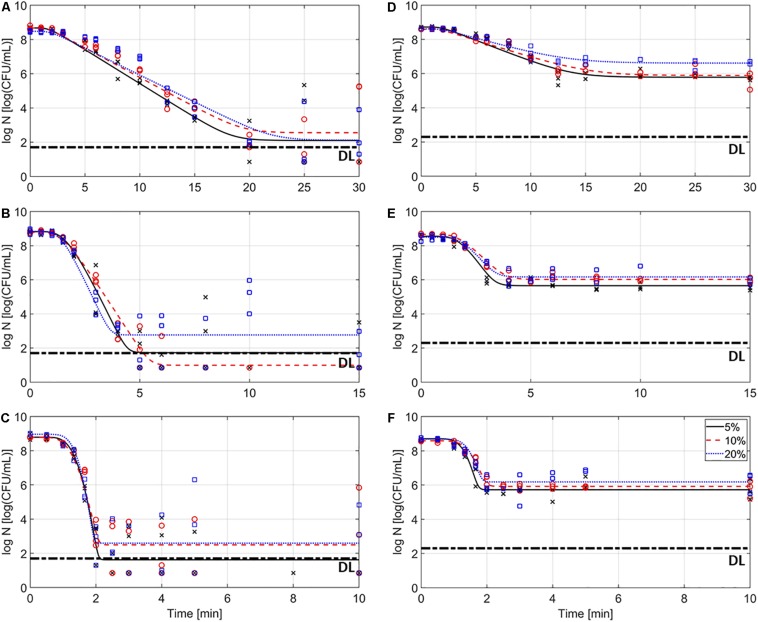
Inactivation kinetics of *Listeria monocytogenes* in the emulsion **(A–C)** and gelled emulsion **(D–F)** model systems with different fat content at temperatures of 59 **(A,D)**, 64 **(B,E)**, and 69°C **(C,F)**. Symbols (x, o, and □, for 5, 10, and 20% fat, respectively) correspond to the experimental data and lines correspond to the model fit of the Geeraerd et al. (2000) model. The detection limit (DL) is also indicated.

Figure 2 represents the inactivation of *L. monocytogenes* over the course of the thermal treatments at 59 (A, D), 64 (B, E), and 69°C (C, F) in the model systems with different fat content (i.e., 5, 10, and 20%), for the emulsions (A–C) and gelled emulsions (D,E), respectively. In this figure, the (non-isothermal) inactivation model of Geeraerd et al. (2000) was fitted to the experimental data. Table 5 shows an overview of the obtained inactivation parameters [i.e., *k*_max_(*T*_ref_) and *z*-value] and mean squared errors (MSE) obtained from the global model fit for each of the model systems. The influence of food matrix fat content on thermal inactivation was investigated by comparing the maximum specific inactivation rate *k*_max_ [i.e., calculated in function of temperature using the obtained value for *k*_max_(*T*_ref_), according to Eq. 3] and the obtained log reductions among the different conditions. Based on the MSE values and Figure 2, it can be observed that the model fit was best for the gelled emulsion systems. The relatively high MSE values for the emulsion systems, however, were mainly caused by the high variation in the data points in the tailing phase of the inactivation. Since this variation was less in the log-linear inactivation phase (i.e., related to *k*_max_), only the estimated log reductions were significantly affected by the accuracy of the model fit.

**TABLE 5:** Maximum specific inactivation rates at the reference temperature *k*_max_(*T*_ref_), *z*-values and mean squared errors (MSE) obtained from the global fit procedure for the emulsion and gelled emulsion model systems with different fat content.

	Emulsions (%fat)			Gelled emulsions (%fat)		
		
Parameter	5	10	20	5	10	20
*k*_max_(*T*_ref_) (1/min)	2.65 ± 0.00	1.96 ± 0.00	2.79 ± 0.00	2.01 ± 0.00	1.40 ± 0.00	1.73 ± 0.00
*z* (°C)	6.05 ± 0.01	6.57 ± 0.00	5.32 ± 0.01	5.53 ± 0.00	5.83 ± 0.00	5.04 ± 0.01
MSE	3.05	3.34	4.80	0.35	0.23	0.43

#### Maximum Specific Inactivation Rate

**FIGURE 3:**
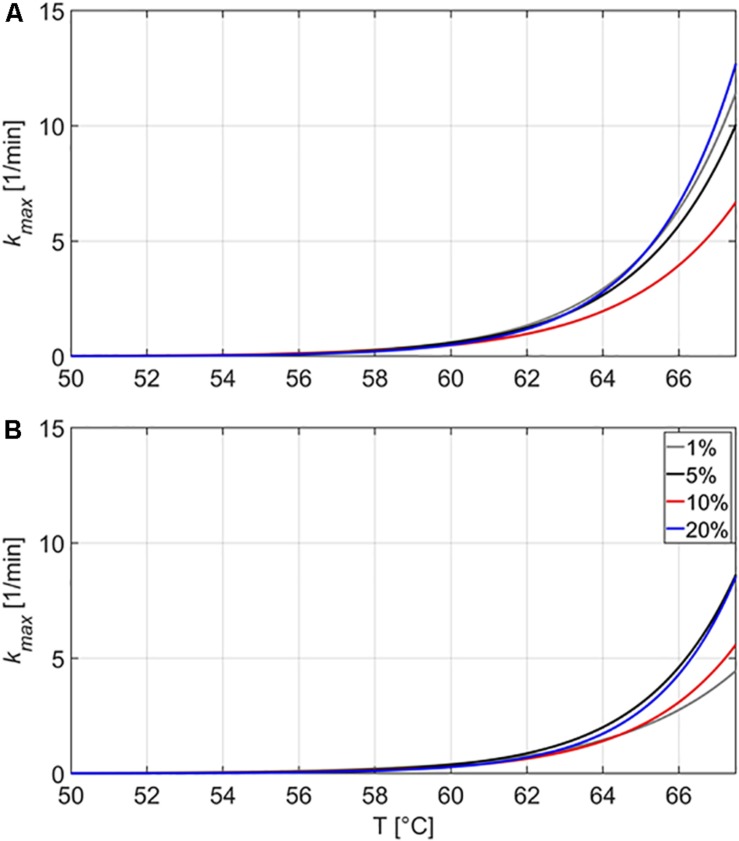
Estimated maximum specific inactivation rate *k*_max_ (1/min) in function of the inactivation temperature, according to the inactivation model of Geeraerd et al. (2000) for the thermal inactivation of *Listeria monocytogenes* in the emulsion **(A)** and gelled emulsion **(B)** model systems with different fat content. Data obtained from Verheyen et al. (2019a) was used to calculate *k*_max_ for the systems containing 1% fat.

Figure 3 presents the *k*_max_ values in the emulsion and gelled emulsion systems with different fat content as a function of the inactivation temperature. In order to have a more complete view on the influence of the fat content, *k*_max_ values for emulsion and gelled emulsion systems containing 5, 10, and 20% of fat obtained in the current study were also compared to *k*_max_ values for the systems containing 1% fat, as provided by Verheyen et al. (2019a). It should be noted that *k*_max_-values at a certain temperature cannot be directly linked to one of the inactivation experiments at 59, 64, or 69°C. Figure 3 shows a general temperature-dependency of *k*_max_ for each model system, calculated on the basis of the three different inactivation experiments. In addition, for the experiments conducted at 69°C, the temperature measurements showed that most of the inactivation occurred before the core temperature of the model systems reached 67.5°C (results not shown). Since the inactivation model was not fitted to inactivation data at higher temperatures and *k*_max_ values at this temperature would not represent any practical relevance, only temperatures up until 67.5°C are therefore shown in Figure 3.

In the emulsions, the maximum specific inactivation rate *k*_max_ decreased with increasing fat content until a temperature of approximately 60°C. At higher temperatures, *k*_max_ in Em20 increased at a higher rate than in the other systems, surpassing *k*_max_ of Em10, Em5, and Em1 at approximate temperatures of 60, 63, and 65°C, respectively. In the gelled emulsions, the maximum specific inactivation rate *k*_max_ also decreased with increasing fat content at lower temperatures. For GE20, *k*_max_ of GE10 and GE1 were surpassed at approximate temperatures of 61 and 63°C, respectively. For GE10, *k*_max_ of GE1 was surpassed at approximately 64°C. Figure 3 also shows that the maximum specific inactivation rate *k*_max_ was always lower in the gelled emulsions than in the respective emulsion-counterparts (i.e., at one temperature for a certain fat content). This last finding confirms the conclusion of Verheyen et al. (2019a) concerning the influence of the nature of the food matrix on thermal inactivation kinetics of *L. monocytogenes*, stating that the presence of a gelled matrix results in lower *k*_max_ values.

Results for *k*_max_ in Em1 (i.e., 0.419 ± 0.001 and 2.928 ± 0.004 1/min at 59 and 64°C, respectively) and GE1 (i.e., 0.292 ± 0.000 and 1.452 ± 0.001 1/min at 59 and 64°C, respectively) were also compared to those obtained in the similar liquid and aqueous gel systems without fat (Verheyen et al., 2019a). In the liquid system, *k*_max_ values were equal to 0.421 ± 0.001 and 2.334 ± 0.012 1/min at 59 and 64°C, respectively. Consequently, the trend of decreasing *k*_max_ with increasing fat content at lower temperatures was confirmed at 59°C in the viscous systems (i.e., liquid without fat and the different emulsions), although the difference between the liquid system and Em1 was rather small due to the limited fat content difference. At 64°C, *k*_max_ in the liquid system was lower than in Em1. Since this difference in *k*_max_ was also higher than would be expected based on the small compositional difference of 1% fat, the presence of an “emulsion structure” seems to result in an increased *k*_max_. In the aqueous gel, *k*_max_ values were equal to 0.246 ± 0.000 and 1.059 ± 0.000 1/min at 59 and 64°C, respectively. In gelled food matrices, the presence of an “emulsion structure” therefore seems to result in an increase in *k*_max_ at all temperatures.

In general, the maximum specific inactivation rate *k*_max_ seems to be complexly related to food matrix fat content, the nature of the food matrix (i.e., viscous or gelled), and the inactivation temperature. At lower temperatures, the protective effect (i.e., lower *k*_max_) of higher fat content could be related to the distribution of the cells inside the food matrix. Confocal laser scanning microscopy experiments in the current set of model systems revealed that *L. monocytogenes* cells grew preferably at the fat-water interface in emulsion and gelled emulsion systems, a trend which became more clear with increasing fat content (Verheyen et al., 2019b). Since the thermal conductivity of sunflower oil is considerably lower than the thermal conductivity of water (as previously mentioned in section “Thermal Conductivity”), there can be a short time period during which the *L. monocytogenes* cells experience a lower temperature when they are situated close to the fat phase. Alternatively, the decrease in *k*_max_ with increasing fat content could have been caused by local differences in water activity (a_w_) which were present among the different model systems. While no significant differences in a_w_ were detected among the emulsions and gelled emulsions with different fat content (Verheyen et al., 2020), *L. monocytogenes* cells would locally be subjected to an environment with lower a_w_ when they are situated at the fat-water interface. This local decrease in a_w_ results in lower heat transfer rates, in turn making the cells less susceptible to heat (Senhaji, 1977; Ahmed et al., 1995). The more complex behavior at higher temperatures could be related to the specific microstructural properties of the (gelled) emulsions. In the emulsions, the influence of fat content on *k*_max_ was rather limited, with large differences only occurring at 10% fat. As shown in Table 3, the viscosity (i.e., represented by the consistency index *K*) of the emulsions decreases with increasing temperature. The rate at which the viscosity changes with increasing temperature is, however, dependent on the emulsion fat content. For Em1, Em5, and Em20, the consistency index *K* decreased by approximately 35% when increasing the temperature from to 57.5 to 70.0°C. For Em10, *K* only decreased by approximately 12% over the same temperature range. This less prominent decrease in viscosity in Em10 possibly caused the lower *k*_max_ in this system by promoting emulsion stability and/or providing a more favorable *L. monocytogenes* cell distribution which locally lowered heat transfer rates. In the gelled emulsions, Figure 1 showed that the rheological properties of the model systems remained rather constant at temperatures lower than 59°C, with gelled emulsions exhibiting more viscous behavior with increasing fat content. At higher temperatures, however, all model systems started to exhibit more viscous behavior. In this regard, the increase in viscous behavior was most prominent in GE5 and GE20. These increased viscous properties might have caused a faster inactivation due to the local promotion of heat transfer in GE5 and GE20, possibly explaining the larger *k*_max_ values in those systems. In addition, the limited increase in viscous behavior of GE1 might explain the rather low *k*_max_ in this system.

In order to further elucidate the aforementioned phenomena, the influence of the specific microstructural characteristics (e.g., rheology) of (gelled) emulsions with different fat content on *L. monocytogenes* cell distribution at different inactivation temperatures could be investigated by means of confocal laser scanning microscopy following different treatments.

#### Log Reductions

Log reductions obtained at the end of each of the respective treatments (i.e., final sampling point) are provided in Table 6. Similar to the approach for *k*_max_, log reductions for emulsion and gelled emulsion systems containing 5, 10, and 20% of fat obtained in the current study were also compared to those for the systems containing 1% fat, calculated based on the data from the experiments by Verheyen et al. (2019a). As mentioned, the accuracy of the estimated model fits could be affected by the substantial data variation in the tailing phase of the inactivation. Therefore, results concerning the obtained log reductions were critically evaluated.

**TABLE 6:** Log-reductions in *Listeria monocytogenes* obtained in the emulsion and gelled emulsion model systems with different fat content following the longest treatment time at each of the three temperatures, i.e., 30, 15, and 10 min for treatments at 59, 64, and 69°C, respectively.

	Emulsions			Gelled emulsions		
		
Fat content (%)	59°C	64°C	69°C	59°C	64°C	69°C
1	4.68 ± 0.04^A1^	5.09 ± 0.17^A1^	4.18 ± 0.07^A1^	3.13 ± 0.05^D1^	2.89 ± 0.03^C1^	2.92 ± 0.02^C1^
5	6.57 ± 0.04^D^	7.10 ± 0.06^C^	7.16 ± 0.19^C^	2.93 ± 0.02^C^	2.90 ± 0.13^C^	2.98 ± 0.07^C^
10	6.12 ± 0.12^B^	7.72 ± 0.04^D^	6.29 ± 0.03^B^	2.70 ± 0.01^B^	2.64 ± 0.06^B^	2.67 ± 0.04^B^
20	6.35 ± 0.05^C^	6.08 ± 0.17^B^	6.37 ± 0.07^B^	2.01 ± 0.02^A^	2.33 ± 0.20^A^	2.51 ± 0.07^A^

*^1^Calculated based on data from Verheyen et al. (2019a). For the different fat contents, values bearing different uppercase letters are significantly different (*P* ≤ 0.05).*

In the emulsions, log reductions were the lowest in Em1 at all temperatures. The relation between the observed log reductions in Em5, Em10, and Em20 was dependent on the treatment temperature. At 59°C, the log reduction was the largest in Em5, followed by Em20 and Em10. It can, however, be observed in Figure 2A that these significant differences in log reduction were probably caused by the variation in the experimental data and do not represent any practical relevance. At 64°C, the log reduction was the largest in Em10, followed by Em5 and Em20. For this case, it can be observed in Figure 2B that the inactivation tail Em5 and Em10 was located around or below the DL. Since significant differences between these two systems could therefore not be accurately determined, the log reductions cannot be assumed significantly different. At 69°C, the log reduction was the largest in Em5, while no significant differences were observed between Em10 and Em20. Since cell densities of some of the replicates were below the DL for all conditions, results concerning the log reductions in the emulsion systems should, however, be critically evaluated. When comparing the obtained log reductions (Table 6) and *k*_max_-values (Figure 3) in the emulsion model systems, it becomes clear that conclusions concerning the influence of food matrix fat content on thermal inactivation of *L. monocytogenes* were not similar. Em1 seems to be the only emulsion in which a considerably lower log reduction (i.e., 2–3 log lower compared to the systems with higher fat content) was observed. Consequently, these protective effects of fat content on *L. monocytogenes* which were discussed for *k*_max_, solely affected the rate at which the cells were inactivated. Trends in *L. monocytogenes* log reductions were not largely affected by the inactivation temperature, implying that the direct effect of fat droplets on the bacterial cells was not temperature-dependent. Temperature-dependent differences in thermal conductivity and rheological properties among the different emulsion systems probably only affected local heat transfer rates, but not the thermotolerance of the cells.

In the gelled emulsions, the log-reductions generally decreased with increasing fat content at all temperatures. The only exceptions to this trend were observed between GE1 and GE5 at 64 and 69°C, as no significant differences were observed at these conditions. Furthermore, a considerable resistant cell population, equal to approximately 6 log (as confirmed in Figures 2D,E), was present in all gelled emulsions at all temperatures. This observation implies a protective effect of a gelled food matrix on *L. monocytogenes* cells, which was also reported by Verheyen et al. (2019a). Similar to the emulsion systems, this protective effect was not largely affected by the inactivation temperature. Furthermore, log reductions decreased with increasing fat content, even though significant differences between GE1 and GE5 were only present at 59°C. Similar to the discussion for the emulsions systems, results for log reductions and *k*_max_-values were also compared among the different gelled emulsions. Again, temperature-dependent differences in thermal conductivity and rheological properties, which affected *k*_max_, did not seem to significantly affect the thermotolerance of the cells. Thermotolerance increased with increasing fat content at all temperatures.

Similar to the discussion for *k*_max_, log reductions in Em1 and GE1 were also compared to those obtained in the similar liquid and aqueous gel systems without fat (Verheyen et al., 2019a). Both for the viscous and gelled emulsions, log reductions in systems containing 1% fat were greater or not significantly different compared to the respective systems without fat. Consequently, inducing an emulsion structure seems to result in a decreased thermotolerance of the *L. monocytogenes* cells. The effect of a further increase in fat content depends on the nature of the food matrix (i.e., viscous or gelled), resulting in a further decrease in thermotolerance in viscous systems (i.e., emulsions) and an increase in thermotolerance in gelled systems (i.e., gelled emulsions).

#### Sublethal Injury Assessment

Sublethal injury is defined as “a consequence of exposure to a chemical or physical process that damages but does not kill a microorganism” (Hurst, 1977). In Table 7, SI of *L. monocytogenes* over the course of the treatments for the different emulsion and gelled emulsion systems was quantified by means of the Time-averaged Injured Cells Coefficient (TICC). Similar to the approach used in the previous sections, results from the current study were compared to those for systems containing 1% fat, as obtained by Verheyen et al. (2019a). At 59°C, TICC values decreased with increasing fat content, with the TICC for the systems containing 1% fat being the highest in both emulsions and gelled emulsions. At 64°C, TICC was also the highest in systems containing 1% fat, while differences between the systems containing 5, 10, and 20% fat were rather limited. At 69°C, differences among all systems were rather small, both for emulsions and gelled emulsions. It should, however, be noted that TICC values at 64 and 69°C were rather low in comparison to those at 59°C, implying that the amount of SI was only significant at 59°C.

**TABLE 7:** Time-averaged Injured Cells Coefficient (TICC) of *Listeria monocytogenes* over the course of the thermal inactivation experiments at 59, 64, and 69°C in the emulsion and gelled emulsion model systems with different fat content.

	Emulsions			Gelled emulsions		
		
Fat content (%)	59°C	64°C	69°C	59°C	64°C	69°C
1	8.2^1^	5.3^1^	2.1^1^	3.9^1^	1.6^1^	1.2^1^
5	7.5	1.1	1.9	2.5	0.4	1.3
10	6.9	1.5	0.8	2.0	1.2	0.5
20	3.6	1.3	1.3	1.8	0.5	1.1

*^1^Calculated based on data from Verheyen et al. (2019a).*

When only taking into account the significant SI at 59°C, TICC values decreased with increasing fat content in the emulsions and gelled emulsions, with the TICC for the systems containing 1% fat being the highest. The general trend that SI is less prominent with increasing fat content could be a confirmation of the protective effect (i.e., lower log reduction) of an increased fat content on *L. monocytogenes* cells, as reported by Chhabra et al. (1999). This straightforward trend for the log reductions, however, was in the current study only observed in the gelled emulsions. Therefore, the influence of food matrix fat content in emulsion systems is possibly more closely related to a protective effect against SI due to the locally smaller heat transfer rates.

#### Summary of the Effects of Food Matrix Fat Content on Thermal Inactivation Dynamics

Food matrix fat content was shown to significantly affect *k*_max_, log reductions and SI of *L. monocytogenes*. Apart from the direct effect on the cells, food matrix fat content was also shown to influence both the thermal conductivity and rheological properties of emulsion and gelled emulsion model systems. Within the small-scale (i.e., 1 mL) model systems of the current study, these changes in thermal conductivity and rheology did not significantly alter global heat transfer dynamics in the systems. These induced changes did, however, locally affect the bacterial cells, resulting in changes in *k*_max_. Table 8 provides an overview of all direct and indirect (e.g., rheology, thermal conductivity) effects of food matrix fat content on microbial inactivation dynamics (i.e., thermotolerance, *k*_max_, SI). Overall, the combination of all these effects gives rise to the complex relation between food matrix fat content and microbial inactivation dynamics.

**TABLE 8:** Overview of the direct and indirect effects of food matrix fat on thermal inactivation dynamics of *L. monocytogenes* in emulsion and gelled emulsion model systems.

	Emulsions		Gelled emulsions	
		
Factor	Possible effects	Result	Effect	Result
Presence of (gelled) emulsion structure	Less favorable cell distribution	*k*_max_ ↑ Thermotolerance ↓	Less favorable cell distribution	*k*_max_ ↑ Thermotolerance ↓
Fat content	Direct effect on cells	Thermotolerance ↓ (1–5%) SI ↓ (1–20%)	Direct effect on cells	Thermotolerance ↑ (5–20%) SI ↓ (1–20%)
Thermal conductivity (lower close to fat)	Cells at fat-water interface experience slower temperature increase	*k*_max_ ↓	Cells at fat-water interface experience slower temperature increase	*k*_max_ ↓
Water activity (lower close to fat)	Cells at fat-water interface experience slower temperature increase	*k*_max_ ↓	Cells at fat-water interface experience slower temperature increase	*k*_max_ ↓
Food matrix rheology (in function of temperature)	Decrease in *K* with increasing temperature is less prominent at 10% fat. This may lead to increased emulsion stability and a more favorable cell distribution	*k*_max_ ↓	More prominent increase in viscous behavior with increasing temperature at 5 and 20% fat leads to local promotion of heat transfer	*k*_max_ ↑

### Comparison to Studies in Real Food Products

To the best knowledge of the authors, the current study was the first attempt at a systematic study of the influence of food matrix fat content on thermal inactivation kinetics of *L. monocytogenes* (i.e., using model systems among which the influence of food matrix fat content was isolated). Results of the current study can be compared to those from other studies using real food products, taking into account that results from these studies might have been influenced by compositional, physicochemical, and microstructural variations among the different systems. In addition, the specific *L. monocytogenes* strains used might also have significantly influenced the inactivation kinetics.

Chhabra et al. (1999), investigated the effect of fat content (i.e., 0.0, 2.5, and 5.0%) in homogenized milk systems (i.e., emulsions) on the thermal inactivation of *L. monocytogenes* Scott A at temperatures ranging between 55 and 65°C. According to their findings, an increase in fat content led to a decrease in inactivation rate of *L. monocytogenes*. When increasing the temperature, however, the protective effect of the fat was reduced. A similar trend was observed for the emulsions in the current study over the same fat content range, as lower *k*_max_-values were observed in Em5 than in Em1, although differences became larger at higher temperatures. As mentioned previously, this specific trend was probably caused by the specific microstructural aspects of the model systems used in the current study. Chhabra et al. (1999) also reported decreasing log reductions when increasing the fat content of their emulsions systems over the range of 0–5%, opposite to what was observed in the current study. This difference could have been due to the large sample size of 100 mL used by Chhabra et al. (1999). In these larger sample, the influence of heat transfer inside the systems was probably more important than in the small samples of 1 mL used in the current study. With increasing fat content, a decrease in both conductive (i.e., due to the lower thermal conductivity of fat in comparison to water) and convective (i.e., due to the higher viscosity of systems with higher fat content) heat transfer occurs. Hence, the systems with higher fat content would have been subjected to lower thermal loads than those with lower fat content, possibly explaining the lower log reductions.

Fain et al. (1991) compared the inactivation of *L. monocytogenes* Scott A in lean (2.0%) and fatty (30.5%) ground beef (i.e., gelled emulsions). They found that D values in fatty beef were approximately double the lean beef values (i.e., equivalent to *k*_max_-values which were halved), both at temperatures of 57.2 and 62.8°C. In the current study, the maximum specific inactivation rate *k*_max_ in the gelled emulsions also decreased with increasing fat content at temperatures below 61°C, while the trend at higher temperatures was more complex.

Schultze et al. (2007) investigated the inactivation of *L. monocytogenes* MFS 102 in frankfurter slurries (i.e., emulsions) containing 8.5, 11.0, and 23.0% fat at 60°C. They reported significantly higher *D* values (i.e., lower *k*_max_ values) in systems containing 8.5% fat than in systems containing 11.0 and 23.0% fat. This trend is opposite to the one observed for the emulsions at similar temperatures in the current study, although an increase of *k*_max_ with increasing fat content was also observed at higher temperatures, e.g., from 10 to 20%. Differences could have been caused by the use of different kinds of frankfurter for the preparation of slurries with different fat contents, i.e., reduced fat frankfurters, regular fat frankfurters, and regular fat frankfurters with additional beef tallow for systems containing 8.5, 11.0, and 23.0% fat, respectively. Consequently, the influence of fat content on microbial inactivation was not effectively isolated.

Finally, Kim and Kang (2015) investigated the inactivation at 60°C of a three-strain *L. monocytogenes* cocktail (i.e., ATCC 19111, ATCC 19115, and ATCC 15313) in mixtures of cream and peptone water (i.e., emulsions) with fat contents of 0, 3, 7, and 10%. They reported no significant influence of fat content on log reductions of *L. monocytogenes* (and *Salmonella* Typhimurium and *Escherichia coli*), which is similar to the findings of the current study over the same fat content range at 59°C.

It was demonstrated that the thermal inactivation of *L. monocytogenes* in real food products exhibits certain similarities to the inactivation in the model systems used in the current study, but findings from the current study cannot be directly transferred to the behavior in real food products. The influence of food matrix fat content on the thermal inactivation of *L. monocytogenes* is probably also dependent on other factors which were not effectively isolated in the real food products. In addition, the use of larger sample volumes may have resulted in an increased importance of heat transfer in comparison to the current study. Therefore, future predictive modeling tools in which the influence of food matrix fat content is incorporated should also include factors related to rheology and thermal conductivity in order to take into account the effect of fat content on heat transfer inside the foods.

## Conclusion

In emulsion and gelled emulsion products, the isolated influence of food matrix fat content on the thermal inactivation of *L. monocytogenes* was shown to be largely dependent on other factors which were also temperature-dependent, e.g., the nature of the food matrix, thermal conductivity, rheological properties. The presence of the (gelled) emulsion structure on itself exerts a negative effect on the thermotolerance of the bacteria, even with a very low fat content (i.e., 1%). Among (gelled) emulsions, however, inactivation rates (i.e., *k*_max_) and thermotolerance (i.e., final log reduction) are affected differently by changes in food matrix fat content and inactivation temperature. While inactivation rates can, under certain conditions, be lowered by increasing the food matrix fat content, the thermotolerance of *L. monocytogenes* is not affected similarly, sometimes even being lower at increased fat content. This behavior is most probably related to the cell distribution inside the food matrix which is affected by the (temperature-dependent) rheological properties of the product on the one hand, and the direct effect of the fat droplet presence on the microbial cells (not largely affected by inactivation temperature) on the other. The direct effect of food matrix fat content on thermal inactivation of *L. monocytogenes* can, however, not be directly applied to predict the behavior of the bacterium in real food products. The influence of food matrix fat content on thermal conductivity and rheology, both affecting heat transfer, becomes more important when food products are larger than the small samples used in the current study.

Since the protective effect of fat content on thermal inactivation dynamics of pathogens is important for the design of thermal pasteurization processes in food industry, further research on this effect should be conducted by, e.g., a microscopic characterization of bacterial cell distribution, rheological characterization of different food products, an investigation of the effect on different bacteria. In this regard, the influence of food matrix fat content should always be investigated in combination with the rheological properties of the food product, since the influence of those two factors on microbial inactivation kinetics is largely intertwined. An elucidation of this effect could also lead to an improved accuracy of predictive models for thermal inactivation processes. Findings from the current study concerning the isolated effect of food matrix fat content at a small scale could also serve as a starting point for the development of such models.

## Data Availability Statement

The datasets generated for this study are available on request to the corresponding author.

## Author Contributions

DV, MB, TS, and JV conceptualized the study. DV, MG, MB, TS, and JV worked on the methodology and supervised the study. DV was responsible for software, validation, formal analysis, data curation, writing and original draft preparation, visualization, and project administration. DV, MG, TKS, JR, and VM carried out the investigation. JV was responsible for the resources and funding acquisition. DV, MG, TKS, JR, VM, MB, TS, and JV wrote, reviewed and edited the manuscript.

## Conflict of Interest

The authors declare that the research was conducted in the absence of any commercial or financial relationships that could be construed as a potential conflict of interest.

## References

[B1] Ahmed N. M. Conner D. E. Huffman D. L. (1995). Heat-resistance of *Escherichia coli* O157:H7 in meat and poultry as affected by product composition. *J. Food Sci.* 60 606–610. 10.1111/j.1365-2621.1995.tb09838.x

[B2] Aquerreta Y. Astiasaran I. Mohino A. Bello J. (2002). Composition of pâtés elaborated with mackerel flesh (*Scomber scombrus*) and tuna liver (*Thunnus thynnus*): comparison with commercial fish pâtés. *Food Chem.* 77 47–53. 10.1016/S0308-8146(01)00310-7

[B3] Arancibia C. Castro C. Jublot L. Costell E. Baryarri S. (2015). Colour, rheology, flavour release and sensory perception of dairy desserts. Influence of thickener and fat content. *LWT Food Sci. Technol.* 62 408–416. 10.1016/j.lwt.2014.08.024

[B4] Baghe-Khandan M. S. Okos M. R. Sweat V. E. (1982). The thermal conductivity of beef as affected by temperature and composition. *Trans. ASAE* 25 1118–1122.

[B5] Baka M. Noriega E. Van Langendonck K. Van Impe J. F. (2016). Influence of food intrinsic complexity on *Listeria monocytogenes* growth in/on vacuum-packed model systems at suboptimal temperatures. *Int. J. Food Microbiol.* 235 17–27. 10.1016/j.ijfoodmicro.2016.06.029 27393885

[B6] Baka M. Vercruyssen S. Cornette N. Van Impe J. F. (2017a). Dynamics of *Listeria monocytogenes* at suboptimal temperatures in/on fish-protein based model systems: effect of (micro)structure and microbial distribution. *Food Control* 73 43–50. 10.1016/j.foodcont.2016.06.031

[B7] Baka M. Verheyen D. Cornette N. Vercruyssen S. Van Impe J. F. (2017b). *Salmonella* Typhimurium and *Staphylococcus aureus* dynamics in/on variable (micro)structures of fish-based model systems at suboptimal temperatures. *Int. J. Food Microbiol.* 240 32–39. 10.1016/j.ijfoodmicro.2016.08.004 27627842

[B8] Busch S. V. Donnelly C. W. (1992). Development of a repair-enrichment broth for resuscitation of heat-injured *Listeria monocytogenes* and *Listeria innocua*. *Appl. Environ. Microbiol.* 58 14–20. 1531746 10.1128/aem.58.1.14-20.1992 PMC195165

[B9] Byelashov O. A. Adler J. M. Geornaras I. Ko K. Y. Belk K. E. Smith G. C. (2010). Evaluation of brining ingredients and antimicrobials for effects on thermal destruction of *Escherichia coli* O157:H7 in a meat model systems. *J. Food Sci.* 75 209–217. 10.1111/j.1750-3841.2010.01595.x 20546412

[B10] Chhabra A. T. Carter W. H. Linton R. H. Cousin M. A. (1999). A predictive model to determine the effects of pH, milkfat, and temperature on thermal inactivation of *Listeria monocytogenes*. *J. Food Prot.* 62 1143–1149. 10.4315/0362-028X-62.10.1143 10528717

[B11] Cordioli M. Rinaldi M. Barbanti D. (2016). Investigation and modelling of natural convection and conduction heat exchange: study on food systems with modified starch by means of computational fluid dynamics. *Int. J. Food Sci. Technol.* 51 854–864. 10.1111/ijfs.13039

[B12] Dybowska B. E. (2011). Whey protein-stabilized emulsion properties in relation to thermal modification of the continuous phase. *J. Food Eng.* 104 81–88. 10.1016/j.jfoodeng.2010.11.030

[B13] Echarte M. Conchillo A. Ansorena D. Astiasaran I. (2004). Evaluation of the nutritional aspects and cholesterol oxidation products of pork liver and fish patés. *Food Chem.* 86 47–53. 10.1016/j.foodchem.2003.08.027

[B14] Erdogdu F. Topcam H. Altin O. Verheyen D. Van Impe J. F. Seow T. K. (2018). “Characterization of fish based model food systems for microwave heating modeling,” in *Proceedings of Foodsim 2018*, eds Van Impe J. Polanska M. (Oostende: EUROSIS-ETI), 235–239.

[B15] Fain A. R. Jr. Line J. E. Moran A. B. Martin L. M. Lechowich R. V. Carosella J. M. (1991). Lethality of heat to *Listeria monocytogenes* Scott A: D-value and z-value determinations in ground beef and turkey. *J. Food Prot.* 54 756–761. 10.4315/0362-028X-54.10.756 31051527

[B16] Garre A. Fernández P. S. Lindqvist R. Egea J. A. (2017). Bioinactivation: software for modelling dynamic microbial inactivation. *Food Res. Int.* 93 66–74. 10.1016/j.foodres.2017.01.012 28290281

[B17] Geeraerd A. H. Herremans C. H. Van Impe J. F. (2000). Structural model requirements to describe microbial inactivation during a mild heat treatment. *Int. J. Food Microbiol.* 59 185–209. 10.1016/S0168-1605(00)00362-7 11020040

[B18] Guinee T. P. Auty M. A. E. Fenelon M. A. (2000). The effect of fat content on the rheology, microstructure and heat-induced functional characteristics of Cheddar cheese. *Int. Dairy J.* 10 277–288. 10.1016/S0958-6946(00)00048-0

[B19] Herigstad B. Hamilton M. Heersink J. (2001). How to optimize the drop plate method for enumerating bacteria. *J. Microbiol. Methods* 44 121–129. 10.1016/S0167-7012(00)00241-4 11165341

[B20] Hurst A. (1977). Bacterial injury: a review. *Can. J. Microbiol.* 23 935–944. 329964 10.1139/m77-139

[B21] Juneja V. K. Eblen B. S. Marks H. M. (2001). Modeling non-linear survival curves to calculate thermal inactivation of *Salmonella* in poultry of different fat levels. *Int. J. Food Microbiol.* 70 37–51. 10.1016/s0168-1605(01)00518-9 11759761

[B22] Kim S.-S. Kang D.-H. (2015). Effect of milk fat content on the performance of ohmic heating for inactivation of *Escherichia coli* O157:H7, *Salmonella enterica Serovar typhimurium* and *Listeria monocytogenes*. *J. Appl. Microbiol.* 119 475–486. 10.1111/jam.12867 26043029

[B23] Kotrola J. S. Conner D. E. (1997). Heat inactivation of *Escherichia coli* O157:H7 in turkey meat as affected by sodium chloride, sodium lactate, polyphosphate, and fat content. *J. Food Prot.* 60 898–902. 10.4315/0362-028X-60.8.898 31207811

[B24] Lim T. Uhl J. T. Prud’homme R. K. (1984). Rheology of self-associating concentrated xanthan solutions. *J. Rheol.* 28 367–379. 10.1122/1.549757

[B25] Lopes da Silva J. A. Rao M. A. (2007). “Rheological Behavior of Food Gels,” in *Rheology of Fluid and Semisolid Foods: Principles and Applications*, ed. Rao M. A. (Now York, NY: Springer), 339–401.

[B26] Ma L. Barbosa-Cánovas G. V. (1995). Rheological characterization of mayonnaise. Part II: flow and viscoelastic properties at different oil and xanthan gum concentrations. *J. Food Eng.* 25 409–425. 10.1016/0260-8774(94)00010-7

[B27] Manthey-Karl M. Ostermeyer U. Altınelataman C. Çelik U. Oehlenschläger J. (2014). Chemical composition, cholesterol, trace metals and amino acid composition of different canned fish products produced and sold in Turkey. *J. Fishscicom.* 8 17–26. 10.3153/jfscom.2014003

[B28] Miller F. A. Brandão T. R. S. Teixeira P. Silva C. L. M. (2006). Recovery of heat-injured *Listeria innocua*. *Int. J. Food Microbiol.* 112 261–265. 10.1016/j.ijfoodmicro.2006.04.013 16784792

[B29] Oliveira R. B. A. Baptista R. C. Chincha A. A. I. A. Conceição D. A. Nascimento J. S. Costa L. E. O. (2018). Thermal inactivation kinetics of *Paenibacillus sanguinis* 2301083PRC and *Clostridium sporogenes* JCM1416MGA in full and low fat “requeijão cremoso”. *Food Control* 84 395–402. 10.1016/j.foodcont.2017.08.030

[B30] Pereira C. G. De Resende J. V. De Oliveira Giarola T. M. Pinto S. M. De Abreu L. R. (2013). Thermal conductivity of milk with different levels of moisture and fat: experimental measures and prediction models. *Semin. Cienc. Agrar.* 34 1153–1166. 10.3168/jds.2016-12051 28259413

[B31] Phinney D. M. Frelka J. C. Heldman D. R. (2017). Composition-based prediction of temperature-dependent thermophysical food properties: reevaluating component groups and prediction models. *J. Food Sci.* 82 6–15. 10.1111/1750-3841.13564 27886381

[B32] Pratap Singh A. Yen P. P.-L. Ramaswamy H. S. Singh A. (2018). Recent advances in agitation thermal processing. *Curr. Opin. Food Sci.* 23 90–96. 10.1016/j.cofs.2018.07.001

[B33] Raju C. V. Shamasundar B. A. Udupa K. S. (2003). The use of nisin as a preservative in fish sausage stored at ambient (28 ± 2°C) and refrigerated (6 ± 2°C) temperatures. *Int. J. Food Sci. Technol.* 38 171–185. 10.1046/j.1365-2621.2003.00663.x

[B34] Reiner M. (1926). Über die strömung einer elastischen flüssigkeit durch eine kapillare. *Kolloid Zeitschrift.* 39 80–87.

[B35] Schultze K. K. Linton R. H. Cousin M. A. Luchansky J. B. Tamplin M. L. (2007). Effect of preinoculation growth media and fat levels on thermal inactivation of a serotype 4b strain of *Listeria monocytogenes* in frankfurter slurries. *Food Microbiol.* 24 352–361. 10.1016/j.fm.2006.07.019 17189761

[B36] Senhaji A. F. (1977). The protective effect of fat on the heat resistance of bacteria (II). *J. Food Technol.* 12 217–230. 10.1111/j.1365-2621.1977.tb00103.x

[B37] Sharqawy M. H. (2013). New correlations for seawater and pure water thermal conductivity at different temperatures and salinities. *Desalination* 313 97–104.

[B38] Stoltenberg S. K. Getty K. J. Thippareddi I. L. Phebus R. K. Loughin T. M. (2006). Fat of *Escherichia coli* O157:H7 during production of snack sticks made from beef or a venison/beef fat blend and directly acidified with citric or lactic acid. *J. Food Sci.* 71 228–235. 10.1016/j.desal.2012.12.010

[B39] Tabilo-Munigaza G. Barbosa-Cánovas G. V. (2005). Rheology for the food industry. *J. Food Eng.* 67 147–156. 10.1016/j.jfoodeng.2004.05.062

[B40] Tavman I. H. Tavman S. (1999). Measurement of thermal conductivity of dairy products. *J. Food Eng.* 41 109–114. 10.1016/S0260-8774(99)00079-5

[B41] Turgut A. Tavman I. Tavman S. (2009). Measurement of thermal conductivity of edible oils using transient hot wire method. *Int. J. Food Prop.* 12 741–747. 10.1080/10942910802023242

[B42] Valdramidis V. P. Geeraerd A. H. Gaze J. E. Kondjoyan A. Boyd A. R. Shaw H. L. (2006). Quantitative description of *Listeria monocytogenes* inactivation kinetics with temperature and water activity as the influencing factors; model prediction and methodological validation on dynamic data. *J. Food Eng.* 76 79–88. 10.1016/j.jfoodeng.2005.05.025

[B43] Van Derlinden E. Bernaerts K. Van Impe J. F. (2008). Accurate estimation of cardinal growth temperatures of *Escherichia coli* from optimal dynamic experiments. *Int. J. Food Microbiol.* 128 89–100. 10.1016/j.ijfoodmicro.2008.07.007 18835500

[B44] Vélez-Ruiz J. F. González-Tomás L. Costell E. (2005). Rheology of dairy custard model systems: influence of milk -fat and hydrocolloid type. *Eur. Food Res. Technol.* 221 342–347. 10.1007/s00217-005-1174-8

[B45] Verheyen D. Baka M. Akkermans S. Skåra T. Van Impe J. F. (2019a). Effect of microstructure and initial cell conditions on thermal inactivation kinetics and sublethal injury of *Listeria monocytogenes* in fish-based food model systems. *Food Microbiol.* 84:103267. 10.1016/j.fm.2019.103267 31421789

[B46] Verheyen D. Xu X. M. Govaert M. Baka M. Skåra T. Van Impe J. F. (2019b). Food microstructure and fat content affect growth morphology, growth kinetics, and preferred phase for cell growth of *Listeria monocytogenes* in Fish-based model systems. *Appl. Environ. Microbiol.* 85:e707–e719. 10.1128/AEM.00707-19 31175191 PMC6677851

[B47] Verheyen D. Baka M. Glorieux S. Duquenne B. Fraeye I. Skåra T. (2018). Development of fish-based model systems with various microstructures. *Food Res. Int.* 106 1069–1076. 10.1016/j.foodres.2017.12.047 29579900

[B48] Verheyen D. Bolívar A. Pérez-Rodríguez F. Baka M. Skåra T. Van Impe J. F. (2020). Isolating the effect of fat content on *Listeria monocytogenes* growth dynamics in fish-based emulsion and gelled emulsion systems. *Food Control* 108:106874 10.1016/j.foodcont.2019.106874

[B49] Wendin K. Hall G. (2001). Influences of fat, thickener and emulsiifer contents on salad dressing: static and dynamic sensory and rheological analyses. *LWT Food Sci. Technol.* 34 222–233. 10.1006/fstl.2001.0757

[B50] Wilson P. D. G. Brocklehurst T. F. Arino S. Thuault D. Jakobsen M. Lange M. (2002). Modelling microbial growth in structured foods: towards a unified approach. *Int. J. Food Microbiol.* 73 275–289. 10.1016/S0168-1605(01)00660-2 11934035

